# The rapid effects of sleeve gastrectomy on glucose homeostasis and resolution of diabetes mellitus

**DOI:** 10.1002/edm2.182

**Published:** 2020-08-30

**Authors:** Emre Bozkurt, Cemal Kaya, Sinan Ömeroğlu, Onur Güven, Mehmet Mihmanlı

**Affiliations:** ^1^ Deparment of General Surgery University of Health Sciences Sisli Hamidiye Etfal Education and Research Hospital İstanbul Turkey; ^2^ Present address: Department of Gastrointestinal Surgery University of Health Sciences Kartal Koşuyolu High Speciality Educational and Research Hospital Istanbul Turkey

**Keywords:** diabetes mellitus, laparoscopic sleeve gastrectomy, obesity, resolution of diabetes

## Abstract

**Aims:**

Type 2 diabetes caused by obesity is increasing globally. Bariatric surgical procedures are known to have positive effects on glucose homeostasis through neurohormonal action mechanisms. In the present study, we aimed to investigate the factors influencing glucose homeostasis independent of weight loss after the laparoscopic sleeve gastrectomy (LSG).

**Methods:**

Patients who underwent LSG for morbid obesity in a 3‐year period were evaluated. Data on demographics, clinical characteristics (duration of diabetes, resected gastric volume, antral resection margin) and laboratory parameters (preoperative and postoperative blood glucose on fasting, preoperative HbA1c levels and first‐year HbA1c levels) were retrospectively reviewed. Effect of patients' body mass index (<50 kg/m^2^, ≥50 kg/m^2^), first‐year excess weight loss (EWL%) rates, age (≥50 years, <50 years), duration of diabetes (≥5 years, <5 years) and antral resection margin (≥3 cm, <3 cm) on postoperative blood glucose profile and diabetic resolution status were investigated.

**Results:**

Total of 61 patients constituted the study group. There were 40 female and 21 male patients with an average age of 43.8 ± 10.5 years (19‐67 years). Preoperatively, mean BMI, blood glucose levels and HbA1c were 48.8 ± 8.5 kg/m^2^, 133.6 ± 47.4 mg/dL and 7.4 ± 1.1, respectively. The mean blood glucose level at the postoperatively 5th day was 88.0 ± 16.3 mg/dL (median: 84 mg/dL) (*P* < .001). Fifty‐nine out of 61 patients improved their glycaemic control.

**Conclusions:**

It is noteworthy that LSG can control blood glucose levels in short term after surgery regardless of weight loss. Therefore, LSG should be preferred at earlier stages in the treatment of obesity‐related T2DM in order to prevent T2DM‐related complications.

## Graphical abstract

Laparoscopic sleeve gastrectomy can control blood glucose levels in short term after surgery regardless of weight loss. Therefore, laparoscopic sleeve gastrectomy should be preferred at earlier stages in the treatment of obesity‐related T2DM, rather than last resort.

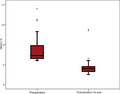

## 1 INTRODUCTION

Obesity‐related type 2 diabetes mellitus (T2DM) is a global ever‐growing health problem, a significant burden on healthcare systems and healthcare costs.^1^ Obesity is an important factor in the onset of T2DM. Therefore, weight loss has a therapeutic importance for success in T2DM treatment. In these patients, lifestyle changes and medications are the main factors for controlling the diabetes. Problems in compliance with long‐term drug use and maintaining weight loss limit the role of these treatment methods.^2^

The development of minimally invasive laparoscopic techniques has provided immense progress in bariatric surgery, and many randomized controlled trials have shown that bariatric surgery reduces cardiovascular risk factors along with successful glycaemic control.^3^, ^4^ Operative results are remarkable in patients with long‐term uncontrolled T2DM.^5^ Similarly, a randomized controlled study conducted by Schauer et al^5^ demonstrated that bariatric surgery in combination with medical therapy is more effective than medical therapy alone for improving T2DM outcomes for patients with body mass index (BMI) from 27 to 43 kg/m^2^.

Out of a host of bariatric operations for morbid obesity, laparoscopic sleeve gastrectomy (LSG) has been the most frequent surgical procedure worldwide. LSG involves removing almost all of the fundus and creating a tube‐shaped stomach with a capacity of 60‐100 mL, which in turn limits the capacity for food intake. LSG was originally defined as a first step of biliopancreatic diversion with duodenal switch (BPDDS), but today it is preferred as a well‐tolerated stand‐alone bariatric surgical procedure.^6^, ^7^

We noticed that in patients with obesity‐related diabetes, blood glucose levels decreased in the first days after LSG. Therefore, we performed a retrospective analysis documenting the improved blood glucose levels after LSG, which was observed before losing significant weight in 61 morbidly obese patients with T2DM. The main focus of our study is to reveal the effect of LSG on glucose homeostasis in the very early postoperative period and to investigate the factors that may cause this effect.

## 2 MATERIALS AND METHODS

Data of obese patients who underwent LSG between 2013 and 2016 at a tertiary referral hospital in Istanbul/Turkey were reviewed. The study protocol was approved by the Local Institutional Review Board of University of Health Sciences Sisli Hamidiye Etfal Education and Research Hospital (approval code 877). Informed consent was obtained from all subjects, and all methods were carried out in accordance with the relevant guidelines and regulations of institutional review board.

### 2.1 Study population and design

Sixty‐one morbidly obese patients with a diagnosis of T2DM were included in the study. The patients were included in this study if the following criteria were met: (a) BMI > 40 kg/m^2^, (b) uncontrolled T2DM after 6 months of medical therapy (HbA1c > 6.5%), and (c) the absence of contraindications for bariatric surgery. Exclusion criteria were as follows: (a) history of duodenal or proximal jejunal intervention, (b) patients with malabsorptive syndrome, and (c) patients who did not attend recall visit or lost to follow up. Data related to patients’ demographics (age, sex, BMI), laboratory results (blood glucose level and HbA1C level) and duration of T2DM were recorded. HbA1c level below 6.5% without the need of antidiabetic treatment for 1 year or blood glucose level of fasting below 100 mg/dL was considered as diabetes resolution.^8^

The parameters used for effectiveness of LSG and weight loss were BMI and excess weight loss ratio (EWL%).

Preoperative blood glucose levels and serum HbA1c, fasting blood glucose and HbA1c level at postoperative 1st, 2nd, 3rd, 4th, 5th days and 1th, 3rd, 6th, 9th and 12th months were recorded. First‐year EWL% and surgical findings were also recorded. Effects of patients' BMI (<50 kg/m^2^, ≥50 kg/m^2^), 1st‐year EWL%, age (≥50 years, <50 years), duration of T2DM (≥5 years, <5 years), resected gastric volume (RcGV) and antral resection margin (ARM) (≥ 3 cm, <3 cm) on postoperative fasting blood glucose level and T2DM resolution status were investigated.

### 2.2 Statistical analysis

SPSS Statistics ver. 15.0 (Chicago, SPSS Inc) was used for statistical analysis. While data for continuous variables were reported as mean ± standard deviation (SD), median, minimum and maximum, categorical variables were presented as number and percentages. Friedman test was used for comparison of numerical variables in dependent groups. Wilcoxon test with Bonferroni correction was performed for subgroup analyses. Chi‐square test was performed for comparison of independent group. A statistical *P* value of .05 or lesser was considered significant.

## 3 RESULTS

### 3.1 Study patients

Totally, 61 patients of which 40 were female and 21 were male were included in this study. The mean age of the patients included was 43.8 ± 10.5 years (19‐67 years). Preoperatively, the mean ± SD BMI, blood glucose level and HbA1c of the patients were 48.8 ± 8.5 kg/m^2^ (range: 40.1‐100.9 kg/m^2^), 133.6 ± 47.4 mg/dL and 7.4 ± 1.1% (median: 6.9%), respectively (Table 1).

**Table 1:** Demographics and clinical characteristics of the patients

		Mean ± SD/Min‐Max (Median)	n (%)
Mean Age (y)		43.8 ± 10.5/19‐67 (42)	
Age (y)	<50		41 (67.2)
	≥50		20 (32.8)
Duration of T2DM (y)		5 ± 2.6/1‐14	
Duration of T2DM (y)	<5		31 (50.8)
	≥5		30 (49.2)
RcGV (cc)		1718.7 ± 434.8/1150‐2200 (1600)	
ARM (cm)		3.7 ± 1.8/2‐8 (3)	
ARM (cm)	<3		39 (63.9)
	≥3		22 (36.1)
Height (cm)		164.5 ± 9.0/150‐186 (164)	
Preoperative weight (kg)		132.1 ± 26.2/100‐295 (130)	
Preoperative BMI (kg/m^2^)		48.8 ± 8.5/40.1‐100.9 (46.9)	
Preoperative HbA1c		7.4 ± 1.1/6.5‐11.6 (6.9)	
Preoperative fasting blood glucose level (mg/dL)		133.6 ± 47.4/90‐318 (116)	
1st‐year EWL%		96.6 ± 18.2/61‐175 (95)	
Resolution of T2DM	No		2 (3.3)
	Yes		59 (96.7)

Abbreviations: ARM, Antral resection margin; BMI, Body mass index; RcGV, Resected gastric volume; T2DM, Type 2 diabetes mellitus.

### 3.2 Glycaemic control

**Figure 1:**
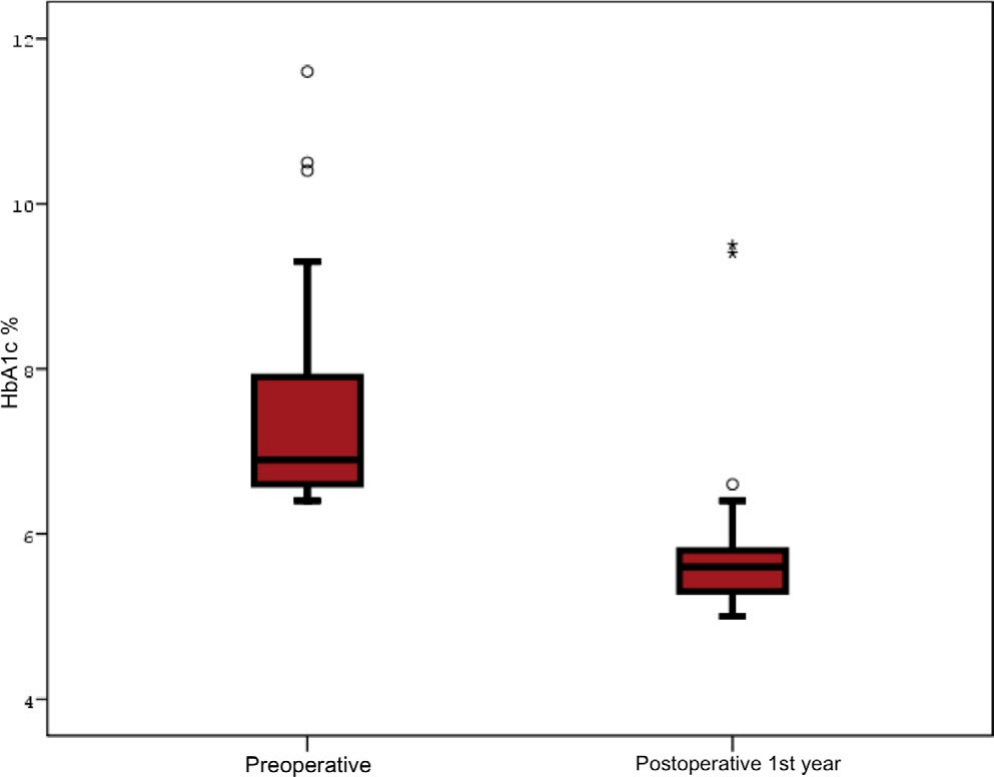
HbA1c levels at preoperative period and postoperative 1st year

The mean blood glucose level on fasting was 88.0 ± 16.3 mg/dL (median: 84 mg/dL) on the 5th postoperative day (*P* < .001) (Tables 2 and 3). The postoperative first‐year blood glucose levels of the patients in the study group are shown in Table 2. Postoperative HbA1c level at the 1st year was 5.7 ± 0.8% (median: 5.6%), and comparison of preoperative and postoperative 1st‐year HbA1c was shown in Figure 1.

**Table 2:** Follow‐up fasting blood glucose level and 1st‐year HbA1c

			Mean ± SD	Min‐Max	Median
HbA1c	1st year		5.7 ± 0.8	5‐9.5	5.6
Fasting blood glucose (mg/dL)	Preoperative		133.6 ± 47.4	90‐318	116
	Postoperative				
		1st day	174.4 ± 43.7	112‐297	165
		2nd day	126.8 ± 43.7	86‐396	117
		3rd day	100.9 ± 27.7	63‐196	94
		4th day	92.3 ± 23.9	64‐179	85
		5th day	88.0 ± 16.3	62‐155	84
		1st month	104.4 ± 38.6	72‐348	94
		3rd month	96.9 ± 27.5	75‐277	91
		6th month	95.2 ± 47.8	65‐413	87
		9th month	90.2 ± 27.7	66‐240	84
		1st year	86.8 ± 23.1	72‐213	82

**Table 3:** The *P*‐values after Bonferroni correction

	*P*
Po day 1 – Pr	<.001
Po day 2 – Pr	.236
Po day 3 – Pr	<.001
Po day 4 – Pr	<.001
Po day 5 – Pr	<.001
Po month 1 – Pr	<.001
Po month 3 – Pr	<.001
Po month 6 – Pr	<.001
Po month 9 – Pr	<.001
Po month 12 – Pr	<.001

Note

Comparison of postoperative fasting blood glucose with preoperative blood glucose.

Bonferroni correction *P* < .0026.

Abbreviations: Po, Postoperative, Pr, Preoperative.

### 3.3 Diabetes medications

The mean T2DM duration of the patients was 5 ± 2.6 years (range: 1‐14 years). There was no statistically significant difference between the groups with diabetes duration ≥5 years and <5 years in terms of T2DM resolution (p:0.238). Fifty‐nine patients ceased all medication for diabetes in the 1 year follow‐up period. None needed increased dosage of antidiabetic therapy. Two patients, whose T2DM did not improve, continued using insulin in the postoperative period, albeit a lower dose of insulin. T2DM was improved in 59 out of 61 patients (Table 1).

### 3.4 Weight loss

Although the mean EWL% at the end of the first month was only 28 ± 8.4%, the rate of decrease in blood glucose was 20% (mean ± SD: 104.4 ± 38.6 mg/dL) in the first month and the blood glucose level reached normal limits in 48 (78.6%) patients during the first month (*P* < .001). At 1st year of follow‐up, the mean ± SD EWL% was 96.6 ± 18.2%, and blood glucose levels decreased by 35% and HbA1c decreased by 23% during this period (Table 2).

We evaluated that T2DM improved in 59 patients (96.7%). Although preoperative HbA1c levels were higher in other 2 patients (3.3%) who had no improvement in T2DM, preoperative HbA1c levels and early postoperative improvement were not statistically relevant. In this study, no difference was found in terms of changes in blood glucose levels, improvement of T2DM and EWL% of patients according to age groups. In terms of surgical technique, we did not find a relationship between the RcGV or ARM and improvement of T2DM (p:0.495 and p:0.531, respectively) (Table 4). No complication was detected related to surgical procedure.

**Table 4:** Effect of demographics and surgical findings on resolution of T2DM

		Resolution of T2DM				
		No		Yes		
		n	%	n	%	
Age (y)	<50	0	0.0	41	69.5	0.104
	≥50	2	100	18	30.5	
Duration of T2DM (y)	<5	0	0.0	31	52.5	0.238
	≥5	2	100	28	47.5	
RcGV (cc)	≤1500	0	0.0	28	47.5	0.495
	>1500	2	100	31	52.5	
ARM (cm)	<3	2	100	37	62.7	0.531
	≥3	0	0.0	22	37.3	
BMI (kg/m^2^)	<50	2	100	40	67.8	1.000
	≥50	0	0.0	19	32.2	

Abbreviations: ARM, Antral resection margin; BMI, Body mass index; RcGv, Resected gastric volume; T2DM, Type 2 diabetes mellitus.

## 4 DISCUSSION

Management of T2DM in obese patients is difficult. HbA1c level below 7% was defined by the current American Diabetes Association as a primary goal of diabetes treatment.^9^ Many studies show that the majority of people with diabetes have difficulty managing their own drug regimens. Even if the recommended drug regimes are followed, these targeted HbA1C levels may not be achieved.^10^, ^11^ Bariatric surgical procedures are accepted as an effective method in the treatment of obesity‐related T2DM.^4^

In our study, laparoscopic sleeve gastrectomy provides dramatic improvement in blood glucose level within the first postoperative week, regardless of weight loss. In parallel with similar studies, there is no correlation between improvement in blood glucose level and EWL% for the early postoperative period.^12^, ^13^ This result supports that the improvement in blood glucose level within early postoperative period may be due to the neurohormonal effects of sleeve gastrectomy.

Prior case series have found that the duration of T2DM prior to surgery affects the remission rates; a less robust improvement in glucose level is seen in those patients who have had T2DM for a long duration and who have high dosage of insulin requirements preoperatively.^5^, ^14^ Contrary to the other published data, in our present study, duration of diabetes or plasma glucose levels did not predict the change in glucose at 1 and 3 months or remission of type 2 DM. A striking result of our study is that 96.7% of patients do not need antidiabetic drugs during the 1 year after surgery. This is a similar result to our previous work.^15^ In a systematic review written by Gill et al,^16^ partial and complete remission rates of T2DM at 13 months of follow‐up after LSG were reported as 26.9% and 66.2%, respectively. However, in this study, it is not clear which mechanisms the difference between diabetes resolution rates depends on.

It has been recently reported that BMI level in the preoperative period did not predict T2DM remission rate postoperatively. T2DM remission occurs without significant weight loss as known by bariatric surgeons.^17^ In our study, T2DM resolution rates were similar for patients with a BMI ≥ 50 and < 50 kg/m.^2^ Therefore, while BMI cannot be used as a measure of metabolic abnormalities, the concept of considering the cut‐off value of BMI as a strict criterion for potential effects of bariatric surgery on T2DM resolution is also controversial.^18^

In this study, the rate of weight loss did not correlate with remission of T2DM. This result concurs with recent studies reporting 84.2% T2DM resolution without significant change of %EWL and fat distribution.^19^, ^20^ This remission can be explained by additional factors known to improve glucose homeostasis. Sista et al^21^ stated that LSG lowers blood glucose level through hormonal mechanisms in the early postoperative period, while preserving this improvement with weight loss effect in the late postoperative period. Lowering of blood glucose level before significant weight loss is a similar finding to other studies in the literature of gastric bypass surgery. Hormonal changes occurring with the removal of the fundus reveal the importance of the stomach in glucose metabolism.^22^ In the late postoperative period after bariatric surgery, the most effective factor on glucose homeostasis is the breaking down of insulin resistance related with weight loss, which lead beta cells to rest and recover.

The first metabolic event seen after LSG is the reduction in the plasma concentration of ghrelin due to the removal of the ghrelin‐secreting cells in the fundus. Increasing resected gastric volume (RcGV) suggests that the more ghrelin‐secreting cell is resected and the more plasma ghrelin level will decrease. Therefore, a larger RcGV is thought to reduce peripheral insulin resistance and have a regulatory effect on insulin release. Although some authors stated that RcGV > 1200 mL was more effective on T2DM resolution,^23^ in our study, no statistically significant difference was detected between groups with RcGV > 1500 mL and ≤1500 mL in terms of blood glucose homeostasis. The lowest RcGV in our study was 1150 mL. Similar to our study, Singh et al^24^ reported a T2DM resolution rate of 82.9%, regardless of the amount of RcGV with a cut‐off of 1700 mL in their prospective study.

Laparoscopic sleeve gastrectomy is a purely restrictive operation with no malabsorptive effect. Improvement in blood glucose levels after malabsorptive procedures occurs within days without significant weight loss.^17^ Although this is not observed in restrictive procedures, review of the literature has shown that LSG has a comparable T2DM amelioration rate in the early postoperative period at rates similar to RYGB.^15^, ^16^, ^25^

While endocrinologists recommend nutritional regulation and strict diet at the initial stage of the disease to prevent catastrophic consequences that can be observed if T2DM is not controlled, surgical intervention performed at early stages of T2DM is likely to be more effective. Since LSG has positive effects on T2DM unrelated with weight loss in the early period, it should be considered as a treatment option in order to prevent T2DM‐related microvascular and macrovascular complications in obese patients in the early period of the disease. The key positive effects of LSG on T2DM independent of weight loss has led to the development of interventional T2DM treatments that do not cause weight loss. In addition, at later stages, LSG also has a positive effect on the recovery of pancreatic beta cell functions with its weight loss effect.

Although fulfilling the sample‐size requirement, a relatively small number of patients were the main limitation of this study. Despite this, we conclude that LSG should be considered as a potential primary treatment option in patients with diabetes.

## CONFLICT OF INTEREST

The authors declare no conflict of interest.

## AUTHORS CONTRIBUTION

EB, CK, SÖ, OG and MM conceived and designed the study; involved in data collection and processing; analysed and interpreted the data; and wrote the manuscript. EB and CK performed literature search. EB, CK and MM critically reviewed the manuscript.

## Data Availability

There are no new data associated with this manuscript.
